# The Third Molar, Wisdom Teeth, or Dens Sapientiæ

**Published:** 1889-09

**Authors:** H. N. Holmes


### The Third Molar, Wisdom Tooth, or Dens Sapientiæ.

BY H. N. HOLMES, D.D.S.

Taking this tooth and attempting to present it in its various phases, from its prenatal condition to its post mortem examination and covering the well worn ground of comparative pathological and eruptive exhibitions, we are somewhat forcibly reminded of the view of that noted humorist Bob. Burdette—he has convulsions when he cut his first teeth and as the last one comes through, lo! the dentist is twisting the first one out, and the end of that man's jaw is worse than the first, being full of porcelain and a roof-plate built to hold backberry seeds.

The third molar has been frowned upon by the profession and by the laity for being late in its development and early in its decay. How correct this stand may be we shall attempt to disclose.

### DEVELOPMENT.

Taken during one of the embryonic stages in the development of the teeth, we find that the six posterior permanent teeth in each jaw, three on each side, arises from successive extensions backwards of the back part of the primitive dental groove. During the fourth month that portion of the dental groove which lies behind the last temporary molar follicle remains open, and from it is developed the papilla, the rudiment of the first molar.

The follicle in which it is contained becomes closed by its operculum, and the upper part of the newly formed sac elongates backwards to form a cavity of reserve in which the papilla of the second permanent molar appears at the seventh month after birth. After a considerable interval, during which the sacs of the first and second permanent molars have considerably increased in size, the remainder of the cavity of reserve presents for the last time a series of changes similar to the preceding, and gives rise to the sac and papilla of the third molar, or wisdom tooth, which appears at the sixth year.

Calcification of the permanent teeth commences a little before birth, taking place in the lower jaw prior to the upper, the first molar in five or six months and the wisdom teeth not till after a lapse of twelve years. The wisdom teeth erupt from the seventeenth to the thirty-fifth year; commonly, however, at about the eighteenth year.

SHAPE.

The crown is small and rounded and furnished with three tubercles in the upper, but the lower being considerably larger than the corresponding one above, and in some instances nearly as large as the superior second molar; the crown is proportionally of greater size. They are little if any smaller than the first lower molar which they greatly resemble in crown, having five cusps similarly situated. The roots are generally confluent and curve strongly back toward the angle of the jaw, but in exceptional cases when the jaws are very fully developed (lantern-jawed if you please) there will often be a two-fang implantation and the whole tooth in a state of development compatible with that of a typical molar.

But among the more civilized races it may almost be said to be exceptional for the third molar dentes sapientiæ, or wisdom teeth to be regular either in form or position. So that extreme variability prevails among these teeth. The roots of some are so confluent as to form an abruptly tapering cone, the apex of which is often bent and crooked so that but little vestige of the two or the three roots can be traced, the pulp cavity even being quite single.

COMPARATIVE.

It is stated in Professor Owen's "Odontography" that although the wisdom tooth is the smallest of the three molars, the difference is less marked in the Melaman than in the Caucasian races, adding also that the triple implantation of the upper, and double implantation of the lower is constant in the former races.

More extended observations have overthrown this statement as a positive dictum to be accepted without exception, but it may nevertheless be taken as expressing a general truth.

In passing from the highest of the apes to the lowest of mankind there is a sudden change in the character of the dentition; but while it can not but be admitted that there is a gap, yet the differences are rather of degree than of kind. Of the anthropoids the gorilla approaches most nearly to man except in dentition, the teeth of the lower jaw perhaps showing the nearest relationship, the third molar attaining a very large size. In the male chimpanzee and orang the wisdom tooth appears before the canine, whereas, in the female, this sexual weapon follows the second, but precedes the third molar. This sexual difference in the canine teeth is well marked in all the members of the branch of the ape family, and its later eruption in the male is explicable both upon the ground that being a sexual weapon, it is not needed prior to the attainment of sexual maturity and also being of a very large size its formation might be expected to take a longer time. No such difference pertains to the milk dentition in which the order of eruption is exactly that met with in man. The coronal aspect and root attachment vary but little from that of man except that the surface of the lower molar is marked by that finely wrinkled pattern which is common to all the unworn teeth of the orang.

It is generally said in man the molars decrease in size from before backwards; that is to say that the first molar is the largest, while in the anthropoid apes the contrary is the case.

While this is on the whole true, it requires some qualification; thus in certain lower races, such as the Australian blacks, the second and third molars are not smaller than the first, but in the chimpanzee the same thing may be said.

One of the particulars in which the lower races of mankind differ from that of the higher is the ample space in which the wisdom tooth has to develop and range itself with the other teeth. It is a characteristic upper or lower molar, the pattern of its grinding surface (quadri-cuspid) if it be an upper quinquicuspid, if it be a lower tooth and the disposition of its roots corresponding with the first and second molars, which do not greatly exceed it in size.

Specimens of negro skulls may be found in which there is scanty room for the wisdom tooth and in which consequently it is a little stunted in the development; on the other hand, plenty of well-formed and well-placed wisdom teeth may be picked out of European mouths, though as a rule the wisdom tooth is much smaller than the other molars, does not bear the characteristic cusps and grooves, has its roots connate and it is not very infrequently a mere rudimentary peg.

The stunted development of the wisdom tooth would seem to be a consequence of want of space during the formative period; the upper wisdom tooth is especially apt to be cramped in this way.

There is some little evidence that the wisdom tooth is in process of disappearance from the jaws of civilized races; in anthropoid apes the wisdom tooth is nearly or quite as large as the other molars, and shows no variability, whilst it comes into place almost simultaneously with the canine; in the lowest races of mankind the wisdom tooth appears to vary but little, is of large size and is seldom misplaced in highly civilized races, it is very variable in size, form and in the date of its appearance, is often misplaced and is not uncommonly quite rudimentary. It seems to be a legitimate inference that a further modification of the race in the same direction will result in the disappearance of the wisdom tooth altogether.

Some exception must, however, be taken to such general statements; thus the Esquimaux not uncommonly have the wisdom teeth smaller and sometimes crowded out of place; and amongst the African races instances on the other hand, of the wisdom teeth being small, and on the other of fourth true molars existing are to be met with. Nevertheless, for the present period, a case in which the wisdom teeth are very small, can hardly be called a typical well-developed American or European mouth.

CARIES.

The coronal surface of the upper teeth form a graceful curve in their natural symmetry with the wisdom tooth at the upper end of the curve, making it almost inaccessible to the bristles of the brush in morning ablutions.

The wisdom teeth universally viewed as being most predisposed to caries, derive such tendency from a twofold direction. Developing at a period when the formative force is losing vigor, these teeth are commonly deficient in the amount that inorganic material which constitutes what might be called the mechanical resistance to the dental organs; in structure they are found, comparatively speaking, loose, while their general resistive power is low; they might, indeed, be likened to the osteophytes which form after bone operations, and which represents so imperfectly the tissue replaced, being found unable to resist antagonisms not all injurious to properly formed tissue. Again, as a local signification is concerned, these teeth, making their appearance at a period when all the others are formed, find so little room in the arch as to render the process of eruption difficult, slow, and in some cases impossible; hence not only is a chronic morbidity engendered, but the face of the tooth is in many instances so long overlaid by an unobserved operculum that a perfect pocket exists, constantly filled by ingesta.

The peculiar lesion which a half erupted wisdom tooth presents on looking into the mouth, is that only the anterior face of the tooth is fairly shown, the other two thirds being overlaid by the integument of the ramus. There may be existing in this tooth, of which only a single cusp is through the gum, caries extending to its pulp cavity or the most aggravated form of periodontitis. It is the common expression that wisdom teeth decay early, that they are not a substantial class of teeth. The fact is that four-fifths of these organs which decay so soon have been destroyed by the operculum of gum under which collect decomposing epithelial scales, and acidity engendered of their disentegration, to corrode the structure of the tooth, thus quickly destroying its integrity. Numbers of cases can be called to mind illustrative of this fact, and if this fleshy lid is dissected off, the proper treatment by the way and the sulci of such teeth examined with a delicate probe, in nine cases out of twelve will caries be found.

An important secondary relation may be recognized from this lesion, namely, that of odonto neuralgia, the diagnosis of which has been as varied and erroneous as the remedies employed. The teeth in such cases probably had not escaped observation, but had been examined and pronounced sound. In such mouths not infrequently will be found this operculum of gum overlying the wisdom tooth, which on being dissected off exposes compound caries. Extraction quite satisfactorily relieves patients of their odonto-neuralgia.

The principal points of attack of decay are in the central depression between the cusps, along the posterior longitudinal fissure, upon the buccal and proximal surfaces.

In buccal decay of much extent it is seldom worth while to attempt treatment by filling. In proximal decay especially upon the inferior, when the tooth is retained, there is danger of causing decay upon the posterior proximal surfaces of the second molar which is likely to extend deep into cervical border, seldom showing upon coronal surface till a large body of the tooth substance is involved, and when filling is required or attempted an almost inaccessible cavity to operate in.

### ABSCESS.

Alveo-dental abscesses are not infrequently associated with the eruption of the wisdom teeth. The arch being so small that when the third molars assert their rights to enter, there is not room enough and hence an intense irritation is caused, generally resulting in an abscess, which discharges about the neck of the tooth. Sometimes, however, they void themselves in other situations, as for example upon the face or neck. An inflammation extending from an ulcerated wisdom tooth may sometimes cause a mistaken diagnosis for tonsilitis and an erroneous treatment for a larger or shorter period. To illustrate another mistake in diagnosis by a case in practice: A patient had suffered some time with heavy dull pain in the right half of the lower jaw, attributed to two teeth, much decayed, but had been treated and filled. Inflammation of a severe character finally developed and in defiance of all treatment, ran on to abscess, which abscess discharged upon the neck.

Lancing and voiding the pus gave some relief, but the sinus continued to discharge and occasion much annoyance as well as deformity. Not till the removal of an erupting dens sapientiæ, a single cusp alone of which presented, did the fistula heal and then in a single week.

In other cases there will be a periodical swelling in the region of the tooth occasioning great pain, coming and going apparently with the changes of the weather, and continuing to occasion a disturbance till the ultimate removal of the offending molar. In the superior maxilla there is found a tuberosity of bone occupied in part by the wisdom tooth and a point of surgical interest, it being not at all uncommon to have necrosis of this portion, the result of an astitis, induced and kept up by an imprisoned dens sapientiæ, standing, as it is seen to do, tubercle like, it is plainly evident that neither deformity nor harm could result from its separation as a sequestrum.

TRISMUS DENTIUM.

But abscesses are not the sole termination. The arch being too small to accommodate the advancing organ, it becomes as a matter of necessity an agent of irritation; inflammations of the most severe nature are thus oftentimes provoked, inducing too commonly trismus and abscess.

The trouble of an individual afflicted with a contracted dental arch are most apt to begin at about the fifteenth or sixteenth year of age. In looking into such a mouth you find the teeth crowded into the most uncomfortable position. The last molar of the lower jaw you will see quite likely jammed into the ramus; while the same tooth of the superior jaw is found occupying the very extreme of the tuberosity of the bone.

At this period unless, fortunately, the teeth are possessed of uncommon resistance, they will be found to be breaking down from aproximal caries, while as the result of such caries, combined with the crowded condition of the roots, the alveo-dental membranes enter into a sub-inflammatory state, and become as ready to take on acute disease as is tinder to respond to a spark. If, then, interference with the elongatory process has been such as to yield these troubles when only twenty-eight teeth have erupted, it is plain that the development of the four dentes sapientiæ must proportionately add to the difficulties. The character of such troubles would lead to the inference that the lesions must be periodontitis, alveolar abscess, stomatitis, astitis, caries, necrosis or trismus.

Case after case of unappreciated local trismus has been reported —of the lesion being referred to this cause and the other cause—the treatment being as various as the diagnosis. Medical men, and withall some members of the dental profession, seem to delight in shrouding such cases in an abstrusely explained mystery, while in reality the cause of such trismus dentium and consequent anchylosis of the train of troubles results from this impacted third molar. The jaw becomes maintained in a fixed position from the rigidity of the muscles closing the jaw, produced by an inflammation, and as a consequence the antagonistic muscles become inadequate to the effort of opening the mouth under the mere influence of volition. Suppurating sore mouth and throat precedes, excessive inflammation attends, and convulsive movements follow affections of this nature.

As soon as the jaws can be separated—the speediest way of which, is by mechanically breaking up the adhesions by introducing forcibly a pine wedge, or another method by galvanism—all this trouble will be found emanating from a wisdom tooth projecting, as it were, from the very angle of the jaw and half covered by an operculum of gum dropping over on it from the ramus. Under treatment (extraction) this lesion disappears, if no inflammation supervenes, as if by magic.

TREATMENT.

It has been demonstrated that the attempted saving by plugging with any of the filling materials is not practicable as a permanent success. Not only is such an operation attended by all the inconveniences that endanger a perfect plug, both from the almost inaccessibility of the cavity and the difficulty of excluding moisture, but the annoyance to the patient and to the operator is mutual.

Filling the wisdom teeth can not therefore be recommended. Of course, this view is taken when all the molars are in position. When any have been extracted, presumably the first molar, then the wisdom tooth is to be preserved from the inroads of decay in the best manner possible. Otherwise, upon the symptoms of dissolution or signs of future trouble, extraction is the most feasible method of relief from what promises to be a never-ending source of irritation.

FORCEPS.

Instruments for extracting this tooth must be so constructed that when the crown of the tooth is grasped in the beak of the forceps it will be directed backwards, thus making the organ roll in a shell-like fashion, as it were, from its socket. For this purpose the bayonet forceps seems to meet the requirement; in any event the application of force must be in the direction of the axis of the root.

The cow horn beak, the right angle beak for small ones, and the so-called Physick's dentes sapientiæ, after the pattern of the late Prof. Physick, are the principal patterns. But this latter instrument; the distinguishing feature of it is its being constructed upon a plan of a double inclined plane, which necessitates its application between the tooth to be extracted and the one directly anterior to it which serves as a fulcrum, not infrequently so crushing the enamel and exposing the dentine as to lead to caries.

When this elevating forceps is used, the beaks or jaws should be at nearly right angle to the handles, so that when the force (closure of the handles) is applied the mouth may not be unnecessarily stretched, as it must be when the pattern is used having the beaks at an angle of 45°. It is presumed that the other molars are in place when attempting the use of this instrument. Care must be exercised when the tooth is deeply imbedded not to force it further into the soft structures. The ordinary key instrument, when slightly and delicately made, answers a very admirable purpose with this class of teeth.

Thus have we considered, in a rather cursory manner, as best our experience served us and the painfully scant literature upon the subject would aid us in our endeavors, the subject of the third permanent molar.

No less conclusion do we come to than this: that if the first molar can be saved and the wisdom tooth is amenable to any of the disturbances already set forth, caries or otherwise, remove the wisdom tooth; otherwise, in the event of the extraction of the first molar at a reasonably early period, and allowing for the others to fill the space, the third molar should be sound to add comfort in mastication and symmetry to the face.

